# Processed meat in the diet: general nutritional profile–protein quality and micronutrients

**DOI:** 10.1093/af/vfaf047

**Published:** 2025-12-18

**Authors:** Mark Wesley Schilling, Sawyer Wyatt Smith, Oladayo Emmanuel Apalowo, Ryen Comey, Shangshang Wang, Thu Dinh

**Affiliations:** Food Science Innovation Hub, Mississippi State University, Mississippi State, MS; Food Science Innovation Hub, Mississippi State University, Mississippi State, MS; Food Science Innovation Hub, Mississippi State University, Mississippi State, MS; Food Science Innovation Hub, Mississippi State University, Mississippi State, MS; Tyson Foods, Springdale, AR; Tyson Foods, Springdale, AR

**Keywords:** DIAAS, micronutrients, nutrition, processed meats, protein quality

Implications

- Meat provides high-quality protein with excellent bioavailability and a complete amino acid profile.
- Meat is highly perishable, but salting, curing, smoking, cooking, drying, fermentation, or a combination of these processes, as well as advanced antimicrobial and antioxidant ingredient solutions, are implemented to slow or inhibit microbial growth. Many of these techniques have been practiced for thousands of years and have been foundational to the safe storage and utilization of animal protein throughout history.
- Processed meats are part of a healthy diet, but they should not be consumed in excess to avoid overconsumption of fat and sodium.
- In developing countries, the availability of processed meats with extended shelf-life helps prevent undernutrition and malnutrition since the digestible indispensable amino acid scores for processed meats are greater than 100%, indicating that processed meats fully meet human amino acid requirements.

## Nutritional Quality of Meat

Processed meats are a significant source of proteins, indispensable amino acids (IAA), A and B vitamins, zinc, phosphorus, iron, and several micronutrients (Table 1). Some public health entities recommend eliminating red meat and processed meats from the diet. Not only are such recommendations not based on sound science, but they also lead to unintended repercussions among vulnerable populations since over 1.5 billion people worldwide suffer from iron deficiency and the heme-iron in red meat is one of the most bioavailable sources of iron in the diet (Kavanaugh et al., 2025).

**Table 1.** Meat products and mineral compositions

Meat product	Macrominerals	Microminerals	Reference
Pork ham (unprepared)	Na: 47 mg P : 199 mg	Fe: 0.85 mg Zn: 1.93 mg	Bohrer (2017)
Pork bacon (cured, unprepared)	Na: 662 mg P : 144 mg	Fe: 0.41 mg Zn: 1.18 mg	
South African processed meats€	Na: 5.4-12.8 (g/kg)	Fe: 13.9–36.1 (mg/kg) Zn: 7–22.6 (mg/kg)	Pretorius et al. (2025)
Hamburger patties	Na: 3.6 (g/kg)	Fe: 31.6 (mg/kg) Zn: 28.6 (mg/kg)	
Frozen beef sausages	Na: 5.2 (g/kg)	Fe: 32.8 (mg/kg) Zn: 24.6 (mg/kg)	
Sausage^±^	Ca: 136.45–400 (mg/kg) K: 610–5800 (mg/kg) Mg: 40–410 (mg/kg)	Fe: 3–197 (mg/kg) Zn: 5–29.77 (mg/kg) Cu: 977–2360 (μg/kg) Cr: 20–100 (μg/kg)	Halagarda and Wójciak, (2022)
Ham^±^	Ca: 100–182 (mg/kg) K: 2475–2749 (mg/kg) Mg: 171–296.5 (mg/kg)	Fe: 4.8–12.1 (mg/kg) Zn: 9.8–27.3 (mg/kg) Cu: 300–1602 (μg/kg) Cr: 55–59.5 (μg/kg) Se: 11–17 (μg/kg)	
Traditional sausages^#^	Mg: 165.07–241.58 (mg/kg) K: 2714.30–3835.15 (mg/kg) Ca: 115.64–178.58 (mg/kg)	Fe: 6.61–9.12 (mg/kg) Zn: 24.26–37.61 (mg/kg) Cr: 32.33–92.93 (μg/kg) Cu: 878.84–1887.11 (μg/kg)	Halagarda et al. (2018)
Conventional sausages^‡^	Mg: 113.19–148.32 (mg/kg) K: 2149.91–2266.55 (mg/kg) Ca: 190.81–506.11 (mg/kg)	Fe: 5.10–8.31 (mg/kg) Zn: 12.19–23.45 (mg/kg) Cr: 25.56–65.11 (μg/kg) Cu: 810.49–1390.78 (μg/kg)	
Broiled beef, trimmed to 0” fat	N/A	Fe: 19.2 (mg/kg) Zinc: 57 (mg/kg)	Kavanaugh et al. (2025)
Cooked, pan-browned Beef, 10% fat	N/A	Fe: 30.8 (mg/kg) Zinc: 68.4 (mg/kg)	
Cooked, grilled Chicken	N/A	Fe: 4.5 (mg/kg) Zinc: 9 (mg/kg)	
Soudjouck	N/A	Cu: 81 (μg/kg) Fe: 1071 (μg/kg) Cr: 87.1 (μg/kg) Mn: 73 (μg/kg) Se: 39 (μg/kg) Zn: 490 (μg/kg)	Demirezen and Uruç (2006)
Salami	N/A	Cu: 84 (μg/kg) Fe: 1276.6 (μg/kg) Cr: 88 (μg/kg) Mn: 57 (μg/kg) Se: 16 (μg/kg) Zn: 450 (μg/kg)	
Sausage	N/A	Cu: 88.3 (μg/kg) Fe: 1564 (μg/kg) Cr: 95.1 (μg/kg) Mn: 100 (μg/kg) Se: 18 (μg/kg) Zn: 600 (μg/kg)	
Pastırma	N/A	Cu: 100.1 (μg/kg) Fe: 1362 (μg/kg) Cr: 89 (μg/kg) Mn: 66 (μg/kg) Se: 46 (μg/kg) Zn: 1590 (μg/kg)	

€ Tinned corned beef, meatballs, French polony, chicken polony, red viennas, and chicken viennas from South Africa.

± Comprise several traditional European meat products.

# Pork meat, pork fat, salt, garlic, spices in various proportions.

‡ Manufactured with the use of food additives and modern production technologies.

## Examples of Processed Meats

### Sausage

Sausage making is a greater than 5000-year-old tradition and is enjoyed globally in various forms, including but not limited to German bratwurst, Cajun Andouille, breakfast patties, and frankfurters. Sausage is a blend of ground, minced, or chopped meat, fat, and seasonings, either stuffed into a casing or formed into patties and then cooked, cured, or smoked prior to consumption. The base of most sausages is high-quality muscle protein, traditionally from pork but also from beef, chicken, turkey, or lamb. The meat is ground and blended with 15% to 30% fat, which contributes to juiciness and flavor. Lipid sources may include lard, beef tallow, poultry fat, or plant-based oils. Sausages may be fresh (raw and cooked before consumption), fully cooked, or dry/fermented (shelf-stable by acidification and dehydration). The digestible indispensable amino acid scores (DIAAS) of sausages are comparable to fresh meat (Fanelli et al., 2024).

### Deli meats

Deli meats are fully cooked and often cured whole-muscle, chunked and formed, or sectioned and formed meat products that are defined as “A ready-to-eat meat or poultry product that typically is sliced, either in the meat plant, foodservice, or grocery store, and typically assembled in a sandwich for consumption.” in accordance with 9 CFR 430.1. While similar to sausage in some respects, their production includes injection and/or tumbling to deliver an even distribution of the ingredients without comminution. Common examples include sliced ham, roast beef, and turkey breast. While historically derived from European charcuterie traditions, modern deli meats are produced with scientific control to ensure quality, safety, consistency, and nutrition. Deli meats retain DIAAS above 100%, similar to unprocessed lean meats. Production typically starts with whole-muscle meats or smaller pieces of meat, which are injected and/or tumbled with a curing brine containing salt, sodium nitrite (if cured), phosphates, and flavorings. Tumbling ensures uniform distribution and extraction of functional proteins. The product is then heat-processed to an internal temperature ≥ 145 °F to 165 °F, depending on the species, ensuring pathogen inactivation, and subsequently chilled, sliced, and packaged.

### Dry-cured hams

Dry-cured hams are artisanal products that are preserved by salting, curing, and air-drying. Examples include Italian *prosciutto di Parma*, Spanish *jamón ibérico*, and US country hams, all with DIAAS >100%. *Prosciutto crudo* and *jamón ibérico* deliver highly digestible, complete amino acids, while also providing zinc, iron, and B vitamins. Like most processed meat products, fat in *jamón ibérico* is rich in monounsaturated fatty acids (up to 55% oleic acid), offering a favorable lipid profile similar to olive oil. Despite being high in salt, dry-cured pork is typically consumed in small, flavor-rich servings or with some form of preparation that removes salt before or during cooking, thus limiting sodium consumption. The transformation from green ham to final product relies on endogenous enzymatic activity, time, and environmental control. Processing begins with rubbing the lean portion of whole hams with salt and nitrate/nitrite and storing the hams under refrigeration conditions for 6 to 8 weeks, which is referred to as the winter season. This is followed by equalization (Spring) and aging under controlled humidity and temperature for months to years (Summer). Natural enzymes and microflora break down proteins and fats over time, developing flavors, umami taste, and desirable texture.

## Functional Ingredients in Processed Meat Examples

### Salt (NaCl)

Salt solubilizes myofibrillar proteins, forming a heat-stable matrix that binds water and fat. Salt addition during the grinding and/or mixing of meat provides negative charges from the chloride ions that extract myofibrillar proteins and disrupts ionic bonds in myofibrillar proteins, allowing for greater solubilization and mixing of other ingredients. This is critical for emulsion stability and the texture of emulsified sausages like frankfurters or bologna. In deli meats, salt is used to solubilize protein, enhance flavor, and inhibit microbial growth. During mechanical tumbling or massaging, salt disrupts ionic bonds in myofibrillar proteins—primarily actomyosin, myosin and actin—allowing them to solubilize and form a protein matrix upon heating. This matrix retains moisture and improves sliceability. Additionally, salt has antimicrobial effects, reduces water activity (aw), and contributes to taste and flavor. Sodium chloride is commonly added such that there is 1% to 2% salt in finished sausages and deli meats. In specialty products such as dry-cured ham, salt is applied (first week and second week) and penetrates the muscle over 6 to 8 weeks of refrigerated storage, initiating osmosis-driven dehydration to an A_w_ of ∼0.88 or lower, during which microbial growth is halted, proteins are denatured, and a unique flavor profile is developed.

### Curing agents

Although salt is the only ingredient required for curing, sodium nitrite and nitrate are often referred to as curing salts and have historically been added to processed meats to inhibit *Clostridium botulinum* growth, while also fixing the characteristic cured flavor and pink color (nitrosyl hemochrome) of cooked, cured sausages. Sodium nitrite also functions as an antioxidant. Usage rates of nitrite and nitrate are listed in 21 CFR 172.175, 21 CFR. 172.170, and 9 CFR 424.21, with legal limits contingent upon the type of product, and erythorbate being included to prevent nitrosamine formation. For example, hams cooked sausage, and bacon have legal usage rates of 200, 156, and 120 ppm, respectively. Clean-label formulations use vegetable-derived nitrite sources such as celery powder. In dry-cured ham, sodium nitrite and nitrate are optional (9 CFR 319.106, 9 CFR 424.21) but can be used to help stabilize the pinkish color early in the curing process, especially in longer-aged hams. Over time, these compounds degrade, becoming undetectable in the final product.

### Phosphates

Monosodium phosphate, tetrasodium pyrophosphate, sodium tripolyphosphate, and sodium hexametaphosphate are used to improve tenderness, increase yield, and increase protein extraction. Phosphates increase pH and enhance ionic strength, thereby improving water-holding capacity (WHC). This allows muscle fibers to retain water during thermal processing. Phosphates also dissociate actomyosin complexes, exposing more protein surface area to bind water and salt. Legal limits (9 CFR 424.21) permit up to 0.5% in the final product, although formulations typically use ∼0.2% to 0.4% due to a soapy flavor that is perceived as the concentration approaches 0.5%.

### Spices and flavorings

These ingredients are integral to cultural and product identity which varies widely by origin—for example, black pepper, paprika, garlic, fennel, sage, black pepper, onion powder, and ginger. They enhance products’ sensory attributes and allow for a great degree of customization. In rare cases, fine particles of seasonings contribute to stabilizing emulsions. European dry-cured hams rarely use additional spices, but US country hams may include a light smoke or pepper rub to differentiate appearance and flavor.

### Sugars

Dextrose, sucrose, and other sugars are added to curing brines to enhance sweetness and balance the saltiness. Since sugars are hygroscopic, they increase water-holding capacity. During thermal processing, sugars help develop colors and flavors through caramelization and Maillard reactions with amino acids.

### Carrageenan

A combination of kappa and iota carrageenan contributes to a homogeneous texture and purge control. A concentration of 0.6% in the final product is sufficient to control purge and allow for thin slicing in deli meats (21 CFR 172.620).

Traditional sausages, deli meats, artisanal dry-cured hams, and other processed meat products play a diverse role in global diets. Although each product undergoes unique processing, they all maintain high-quality, bioavailable protein that supports human nutrition. Misconceptions surrounding ingredients such as salt, nitrites, and phosphates often stem from lack of understanding of their functions. When used within regulatory limits, these ingredients contribute to food safety, shelf-life, flavor, and moisture retention—without degrading protein quality. The USDA FoodData Central database confirms that processed meat proteins remain nutritionally robust and digestible. Cured or cooked meats meet or exceed the protein quality of fresh meat. When consumed in moderation, these foods contribute to a healthy, balanced diet—supporting protein intake, minimizing waste, and honoring centuries of food tradition enhanced by modern food science practices.

## Protein

The international Recommended Dietary Allowance (RDA) for protein is 0.83 g/kg body weight (BW) to meet the minimum indispensable (or essential) amino acid requirements and maintain nitrogen balance (Carbone & Pasiakos, 2019). For healthy adults, two to three meals a day with 25 to 30 g of high-quality protein per meal is ideal for muscle protein synthesis. Animal-derived protein includes all essential amino acids. Cured ham and bacon have 17 to 18 g and 12 to 13 g of dietary protein per 100 g serving, respectively (Bohrer, 2017). Polish sausages contain 11.3 to 24.4 g/100 g protein (Halagarda et al., 2018). Although, raw beef and chicken contain 18 to 20 g of protein per 100 g (Pretorius et al., 2025), which is greater than that of some processed meats, such as canned corned beef and meatballs (8.3 to 13.3/100 g), most processed meats have comparable protein content to similar meat blocks (e.g., raw beef patties vs. frozen beef sausages, raw chicken meat vs. chicken sausage). Processed meats have varying protein contents, depending on the meat blocks that are used in production. Therefore, consumers need to review the nutritional label for their specific dietary needs.

## Lipid

Triglycerides are the predominant lipids in processed meats (9 kcal/g) and aid in the intestinal absorption of fat-soluble vitamins (A, D, E, and K) (Gropper et al., 2022). While the body can synthesize fatty acids and cholesterol via the acetyl-Co A pathway, some polyunsaturated fatty acids like linoleic and α-linolenic acids, which are essential for cell membranes and signaling, must be obtained from the diet. Fish and flaxseed are good sources of these fatty acids. Red meat, poultry, fish, dairy, and eggs supply over half of the unsaturated fat, and the majority of saturated fat and cholesterol in the diet. Fat content in meat is variable, ranging from 4% to 16.2% in various meat products. Processed meats vary in lipid content from practically devoid (close to 0%) in lean deli meat to a maximum allowable amount of 30% in frankfurters and 50% in fresh sausage. However, the majority of frankfurters are approximately 20% fat while fresh sausages generally contain less than 30% fat. Fatty acid composition and cholesterol content of processed meats are similar to the meat blocks from which they are manufactured. The fatty acid composition of meat and poultry is rich in monounsaturated fatty acids, especially a high abundance of oleic acid, similar to olive oil. Saturated fatty acids in processed meats are comprised of a substantial proportion of stearic acid, which is considered neutral in the context of healthy diets. Moreover, in most sausages, dry seasonings (1% to 3%) are typically of plant origin and add a small portion of linoleic and linolenic acids to the finished products.

## Sodium

Processed neat products account for approximately 20% of daily sodium consumption (Fang & Zhu, 2025). The World Health Organization recommends that adults consume less than 2000 mg of sodium (corresponding to under 5 g/day of salt) (Yu et al., 2024). Bohrer (2017) reported that cured bacon contains 662 mg per 100 g of bacon, while tinned corned beef, tinned meatballs, hamburger patties, frozen beef sausages, French and chicken polony, red and chicken Viennas ranged from 363 mg sodium/100 g in some frozen hamburger patties to > 1000 gm sodium/100 g in polonies and Vienna sausages. Excessive sodium intake raises the risk of cardiovascular disease. However, 1000 to 1500 mg of sodium per day is necessary to regulate blood pressure, water transport, tissue osmolality, and nerve cell impulse transmission (Sparks et al., 2018; Tremblay et al., 2024). However, salt reduction or replacement decreases tenderness, flavor, gel strength, and water-holding capacity, as well as increases the risk of spoilage and pathogenic bacteria growth (Wang et al., 2023). Salt is an important ingredient in meat and plays a critical role in the human body, but it is important to be aware of how much salt is being consumed, because too much salt in the diet can be unhealthy, especially for those people who are genetically predisposed to cardiovascular disease. In addition, use of ingredient technology such as replacing a percentage of salt with KCl can help reduce the amount of sodium in processed meats.

## Micronutrients

Micronutrients are essential for bodily functions. They include water-soluble and fat-soluble vitamins as well as trace minerals (required in >100 mg daily (Allen, 2025). Minerals are inorganic elements derived from the earth, categorized as macro or trace. Macro-minerals (calcium, phosphorus, magnesium, sodium, potassium, and chloride) are required in amounts over 100 mg/day. Trace or microminerals (iron, zinc, copper, selenium, chromium, iodine, manganese and molybdenum) are required at between 1 and 100 mg/day, while ultra-trace minerals (including aluminum, arsenic, boron, bromine, nickel, cadmium, germanium, lead, lithium, rubidium, silicon, tin and vanadium) are required in amounts less than 1 mg/day, based on their established essentiality (Gropper et al., 2022). Micronutrient deficiencies, which impact one-third of the global population, have been the focus of interventions in low- and middle-income countries where these deficits are most severe and prevalent (Allen, 2025). Moreover, a recent study revealed that more than fifty percent of preschool-aged children and two-thirds of nonpregnant women of reproductive age globally exhibit micronutrient deficiencies (Stevens et al., 2022).

Several micronutrients are either exclusively found in meat or have higher bioavailability compared to plant sources. Vitamin A (retinol) and B12 are not present in plants, while iron and folic acid from meat (particularly liver) and eggs are absorbed much better than they are from vegetables, indicating that low or no meat intake can lead to nutrient deficiencies (Nohr & Biesalski, 2007). In addition, meat and liver are nutrient-dense foods, with 100 g of low-fat pork providing 1.8 mg iron and 2.6 mg zinc, while pig liver offers 360 mg magnesium, 20 mg iron, and 60 μg selenium, together covering up to 50% of the RDA for several essential nutrients and 100% of vitamin A (Nohr & Biesalski, 2007). Table 1 reports the mineral concentrations in processed meat products.

## Protein Quality in Processed Meats

### Digestibility

Animal-based proteins from processed meat are highly digestible, allowing for excellent amino acid absorption (Ajomiwe et al., 2024). The rapid degradation of protein leads to elevated amino acid concentrations in the bloodstream. An increase in branched-chain amino acids (leucine, isoleucine and valine) are particularly advantageous, since they promote protein synthesis and decrease protein degradation. Digestibility and the availability of critical amino acids are indicators of protein quality, determining nutritional quality. One of the measures of protein quality is the DIAAS, which accurately assesses protein quality by considering amino acid quantity and digestibility, thus aiding dietary recommendations and food product development (Moughan & Wolfe, 2019). The DIAAS is determined using standardized ileal digestibility (SID) values that account for basal endogenous amino acid loss and are equivalent to the true ileal digestibility values required for human food evaluation (Bailey et al., 2020). A DIAAS value above 100% indicates that the test protein provides more than enough of the most limiting amino acid to meet daily needs, whereas a value below 100% means that specific protein provides less than the daily amino acid requirement when consumed at the estimated average requirement for protein (Wolfe et al., 2016). Recent studies have revealed that beef, fish, and animal protein hydrolysates exhibit superior DIAAS compared to plant proteins, with meat offering a concentrated and balanced profile of IAA for human nutrition (Bindari et al., 2018; Reynaud et al., 2021). Soy, wheat, and pea proteins are variable but have DIAAS values of approximately 90% to 95%, <50% and 60% to 80%, respectively (Herreman et al., 2020).

Bailey et al. (2020) determined the DIAAS of salami, bologna, beef jerky, beef ribeye roast (cooked to three degrees of doneness: 56 °C, 64 °C, or 72 °C), raw ground beef, and cooked ground beef for older children, adolescents, and adults. The DIAAS for all proteins, with the exception of cooked ground beef, were >100%. Bologna and 64 °C ribeye roast had the highest DIAAS. Cooking meat to 70 °C increases proteolysis due to heat-induced protein denaturation; however, cooking to an internal temperature of 100 °C causes protein oxidation and the formation of carbonyl side chains in basic amino acids (lysine, histidine, and arginine) and threonine, decreasing ground beef digestibility and protein quality and compromising the bioavailability of essential amino acids, overall leading to a decrease in nutritional value. A similar experiment conducted by Hodgkinson et al. (2018) revealed that the DIAAS for raw, boiled, grilled, pan-fried and roasted beef top-round steak was 97%, 99%, 80%, 98%, and 91%, respectively. Bailey et al. (2020) reported that bacon and ham all had above 100% DIAAS scores, meeting or exceeding the ideal protein for both children and adults. These findings indicate high DIAAS scores for most processed meats (above 100%) and that processed meats are a high-quality protein source; however, the degree of cooking and preparation may negatively impact protein digestibility, which is true for all food proteins as they undergo thermal oxidation during cooking and autoxidation during storage.

### Essential amino acids

The body continuously replaces degraded proteins with newly synthesized ones to maintain protein mass. The main metabolic purpose of essential amino acids is to support protein synthesis (Wolfe et al., 2016). Apart from protein synthesis, amino acids are precursors to purines, pyrimidines, and neurotransmitters, as well as generates nitric oxide, which serves in signaling pathways and physiological processes like vasodilation, neurotransmission, inflammation, and apoptosis (Andrabi et al., 2023; Wolfe et al., 2016). Bailey et al. (2020) demonstrated that the SID of most amino acids did not vary among processed meats like salami, bologna, beef jerky, and cooked ground beef, but was lower than raw ground beef (99.4%), again confirming that cooking may decrease digestibility due to thermal oxidation. Essential amino acid composition of beef jerky (cured with water, salt, brown sugar, sugar, monosodium glutamate, maple sugar, flavorings and sodium nitrite) was totaled at 24.2% and comprised of 3.0%, 2.0%, 2.5%, 4.0%, 4.4%, 0.9%, 2.0%, 2.2%, 0.7%, and 2.6% for arginine, histidine, isoleucine, leucine, lysine, methionine, phenylalanine, threonine, and valine, respectively. This is much greater than raw ground beef with a total of 8.54% essential amino acids, followed by cooked ground beef (11.83%), ribeye roast cooked to an internal temperature of 64 °C (13.71%), ribeye roast cooked to an internal temperature of 72 °C (10.83%), and ribeye roast cooked to an internal temperature of 56 °C (10.72%).

The existing Dietary Reference Intakes (DRIs) specify values for human dietary requirements concerning essential amino acids (EAAs) and are expressed in milligrams per kilogram of BW per day as follows: His: 14; Ile: 19; Leu: 42; Lys: 38; Met + Cys: 19; Phe + Tyr: 33; Thr: 20; Trp: 5; and Val: 24 (Institute of Medicine, 2006). These essential amino acids are not produced de novo by animal cells or are not generated in sufficient quantities for metabolic requirements. Leucine, isoleucine, and valine are branched chain essential amino acids often consumed by athletes or supplemented to healthy neonates with a normal birth weight for increasing lean muscle mass. Arginine augments nitric oxide synthesis, a major vasodilator and an inhibitor of platelet adhesion to blood-vessel walls, which also enhances fertility and improves metabolic profiles. Lysine has been used to treat herpes simplex by inhibiting arginine uptake into the host cell. Tryptophan prevents insomnia by aiding in the synthesis of neurotransmitters: serotonin and melatonin, in the brain while threonine is supplemented to weanling mammals to augment intestinal mucin synthesis and enhance mucosal integrity (Hou & Wu, 2018).

Deficiencies of essential amino acids occur when diets do not provide adequate amounts to humans. A deficiency of an essential amino acid in the diet leads to a progressive increase in the oxidation of other amino acids when dietary intake of amino acids or protein rises (Hou & Wu, 2018). The limited availability of tan essential amino acid restricts the use of other amino acids for protein synthesis, resulting in the degradation of all surplus amino acids in a tissue-specific manner (Hou & Wu, 2018). Essential amino acid deficiencies cause widespread health issues associated with growth and development (growth stunting and impaired cognitive development), neurological, psychological and cognitive disorders and mental health (insomnia, memory loss, headaches), digestive and absorptive disorders (low appetite, vomiting and impaired nutrient absorption and utilization), endocrine imbalance, immune dysfunction, and susceptibility to diseases (Hou & Wu, 2018). Specifically, arginine deficiency contributes to fatigue, developmental delays and impaired immune function. Isoleucine deficiency contributes to muscle wasting and tremors, most commonly in the elderly population. Leucine deficiency presents itself in muscle weakness and wasting, inability to regulate sugar, stunted growth, delayed development, and failure to thrive in children. Lysine and methionine deficiency both cause hair loss, poor growth, appetite loss, fatigue, mood changes, and anemia. Methionine deficiency is also associated with fatty liver, muscle wasting, high cholesterol, and liver damage. Threonine deficiency leads to confusion, fatty liver disease, slower wound healing, agitation, and digestive problems. Valine deficiency presents itself as fatigue, muscle weakness, impaired motor coordination, and cognitive impairments. Essential amino acids deficiencies are extremely difficult for children to overcome, often leading to stunting, undernutrition, and failure to thrive, especially in developing countries.

Moreover, certain amino acids undergo oxidation, which leads to the formation of carbonyl groups. Essential amino acids like histidine, lysine, methionine, threonine, and tryptophan are reactive and can lose bioavailability due to heat, oxidation, or alkaline conditions. Since humans require these basic amino acids, their oxidation considerably reduces the nutritional value of meat. Carbonyls can also interact with the free amino groups of nonoxidized amino acids in proteins to form amide bonds, leading to agglomeration, which inhibits protein digestion (Gatellier et al., 2010; Liu & Xiong, 2000). Phenylalanine and tryptophan are aromatic amino acids required by humans, particularly in cases of phenylketonuria. Free radicals are capable of hydroxylating aromatic amino acids, leading to the formation of dityrosine bridges and protein aggregation, subsequently diminishing the nutritional value of meat. Although cysteine is not an essential amino acid, it may be required due to inadequate production of homocysteine. Cysteine oxidation promotes protein aggregation by forming disulfide bonds. Gatellier et al. (2010) reported that cooking at 65 °C did not alter the level of free thiols, but f concentrations increased at 96 °C when steam was used as the heat source. Moreover, increased free thiols form disulfide bridges, leading to structural changes, protein aggregation, and altered function due to poor nutritional value.

When analyzing lysine in processed foods, Maillard-reacted lysine is nonbioavailable but can revert to lysine during acid hydrolysis (Loveday, 2023). Although cooking can oxidize meat myofibrillar proteins as previously discussed, decreasing pepsin activity and amino acid digestibility (Santé-Lhoutellier et al., 2008), this only occurs at an internal temperature of 100 °C or greater. However, it is important to note that processed meats have abundant water, with the exception of some dry-cured or dehydrated products. Therefore, it is almost impossible for the internal temperature to exceed the boiling point of water.

Processed meats can mitigate essential amino acid deficiencies since all essential amino acids are provided in equitable quantities for biological processes. Meat proteins have excellent digestibility (true fecal digestibility ≥ 90%), enabling the body to effectively absorb and utilize its essential amino acids (Bailey et al., 2020). Meat products include a higher concentration of IAA per serving compared to plant proteins due to a deficient or unbalanced essential amino acid content, hence necessitating smaller quantities to fulfill daily dietary requirements (Tessari et al., 2016). This renders populations that are susceptible to IAA shortages to be more inclined to consume sufficient quantities of essential amino acids. Cured, smoked, or grilled meat products safeguard against deficiencies, owing to their high DIIAAS.

## Importance of Processed Meats in Developing and Third-World Countries

According to the World Health Organization (WHO, 2024), low- and middle-income countries account for more than half of all deaths among children under the age of five due to undernutrition. Undernutrition consists of four main types: wasting (low weight-for-height), stunting (low height-for-age), underweight (low weight-for-age), and deficiencies in vitamins and minerals (Verma & Prasad, 2021; Quamme & Iversen, 2022). While wasting is typically associated with weight loss due to food shortages or infections, stunting is the result of chronic undernutrition, both of which can affect health and cognitive development but are treatable (WHO, 2024). Although meat provides key macro and micronutrients for optimal health, several observational studies have emphasized risks associated with its consumption. For example, in high-income Western nations, studies indicate that high consumption of red and processed meat correlates with marginally increased total mortality rates and chronic diseases (Etemadi et al., 2017; Qian et al., 2020). However, the majority of the studies are epidemiological in nature and potentially confounded by additional risk factors such as smoking, alcohol consumption, genetic predisposition, and obesity. Hence, causation remains unestablished as most studies on red and processed meat intake consist of epidemiologic evidence (prospective and meta-analyses).

Meat processing alters nutritional composition through the inclusion of seasonings and other functional ingredients to improve quality and safety throughout transportation, storage, retail display, and home consumption. Processed meats have incomparable protein quality among all foods. Most micronutrients from processed meats have great bioavailability. The human body readily accepts these natural sources of proteins due to the similarity in amino acid composition. The current body of literature mostly depends on epidemiological data to assess meat consumption risks, using a very flawed approach to calculating hazard ratios, which are confounded with random factors such as genetics, medical history, and lifestyle. Mechanistic studies on health issues mostly employed compounds similar to those released during the digestion and absorption of processed meats without accounting for the impacts of processed meat proteins as a biological source. Depending on specific needs, consumers may need to read the nutritional labels to decide the average daily intake for a specific health outcome. Further processing also serves a unique purpose in ensuring the abundance of muscle foods at an affordable price due to our ability to produce and preserve this highly perishable source of protein. Public health recommendations in general should prioritize the promotion of meat consumption and preservation to combat health disparities such as malnutrition and undernutrition for vulnerable populations.

## About the Authors

Wes Schilling is a William L. Giles Distinguished Professor of meat science, sensory science, and food chemistry at Mississippi State University (MSU), the Director of the Food Science Innovation Hub, and the Reed Family Endowed Professor. Since coming to Mississippi State University, he has developed 5 courses that focus on hands-on experiences and real-world examples that he has experienced through interaction with food companies. Real world examples are related to topics taught through food chemistry, instrumental analysis, and sensory testing principles. His research focuses on sensory science, meat processing (poultry, beef, pork, and catfish), meat quality, flavor chemistry, proteomics and statistical methods. His specific research program on sensory research, broiler meat quality, and relationships between sensory quality, flavor chemistry, and consumer science studies have earned him numerous University and National awards. He has published greater than 200 peer reviewed journal papers since 2003, and more than 125 since 2013. Since 2008, he has also published 18 chapters in textbooks, including 8 in the 6th edition of Principles of Food Sanitation.

**Corresponding author:**  schilling@foodscience.msstate.edu

Sawyer Wyatt Smith is a Ph.D. student at North Carolina State University in the Prestage Department of Poultry Science. He earned his B.S. and M.S. degrees in Food Science and Technology from Mississippi State University. His work focuses on muscle foods products and processing to improve quality, safety, and shelf-life across the food and poultry industry.

Dr. Oladayo Apalowo serves as a postdoctoral associate in the Department of Biochemistry, Nutrition, and Health Promotion at Mississippi State University. Dr. Apalowo’s research synthesizes nutrition, biochemistry, and public health to enhance precision-based therapies. He is enthusiastic in participating in multidisciplinary, population-based research investigating the influence of body composition, dietary patterns, genetic factors, and environmental exposures on the risk of chronic diseases.

Ryen Comey is a M.S. food science student at Mississippi State University in the Department of Biochemistry, Nutrition, and Health Promotion. She earned her B.S. degree in both Biochemistry and Animal and Dairy Sciences from MSU. Her work thus far focuses on helping industry companies improve the shelf-life and quality of their products.

Shangshang Wang is a food scientist at Tyson Foods working with pet food. He earned his Ph.D. in meat science from Mississippi State University.

Thu T. Dinh is a Fellow ­Scientist at Tyson Foods, primarily supporting innovation for the Ingredient Solutions Group and serving as chemistry subject matter expert for Tyson R&D, plants, and other meat and poultry business units within Tyson. He also spent 8 years on faculty in the Department of Animal and Dairy Sciences at Mississippi State University. He has a bachelor’s degree in food engineering from HCMC University of Technology in Vietnam and a Master of Science degree in Food Technology and a Doctor of Philosophy degree in Animal Science, both from Texas Tech University. He is a meat scientist by training and an analytical chemist and flavor chemist by choice. At the end of the day, Thu is proud to call himself a meat man and an educated butcher.
